# Fouling of Polymeric Hollow Fiber Heat Exchangers by Air Dust

**DOI:** 10.3390/ma13214931

**Published:** 2020-11-02

**Authors:** Ilya Astrouski, Miroslav Raudensky, Tereza Kudelova, Tereza Kroulikova

**Affiliations:** Heat Transfer and Fluid Flow Laboratory, Faculty of Mechanical Engineering, Brno University of Technology, Technicka 2, 616 69 Brno, Czech Republic; Ilya.Astrouski@vut.cz (I.A.); Miroslav.Raudensky@vut.cz (M.R.); Tereza.Kroulikova@vut.cz (T.K.)

**Keywords:** polymeric hollow fiber, heat exchangers, heat transfer, fouling

## Abstract

Currently, liquid-to-gas heat exchangers in buildings, domestic appliances and the automotive industry are mainly made of copper and aluminum. Using plastic instead of metal can be very beneficial from an economic and environmental point of view. However, it is required that a successful plastic design meets all the requirements of metal heat exchangers. The polymeric hollow fiber heat exchanger studied in this work is completive to common metal finned heat exchangers. Due to its unique design (the use of thousands of thin-walled microtubes connected in parallel), it achieves a high level of compactness and thermal performance, low pressure drops and high operation pressure. This paper focuses on an important aspect of heat exchanger operation—its fouling in conditions relevant to building and domestic application. In heating, ventilation and air conditioning (HVAC) and automotive and domestic appliances, outdoor and domestic dust are the main source of fouling. In this study, a heat exchanger made of polymeric hollow fibers was tested in conditions typical for indoor HVAC equipment, namely with the 20 °C room air flowing through the hot water coil (water inlet 50 °C) with air velocity of 1.5 m/s. ASHRAE test dust was used as a foulant to model domestic dust. A polymeric heat exchanger with fibers with an outer diameter of 0.6 mm (1960 fibers arranged into 14 layers in total) and a heat transfer area of 0.89 m^2^ was tested. It was proven that the smooth polypropylene surface of hollow fibers has a favorable antifouling characteristic. Fouling evolution on the metallic heat transfer surfaces of a similar surface density was about twice as quick as on the plastic one. The experimental results on the plastic heat exchanger showed a 38% decrease in the heat transfer rate and a 91% increase in pressure drops after eighteen days of the experiment when a total of 4000 g/m^2^ of the test dust had been injected into the air duct. The decrease in the heat transfer rate of the heat exchanger was influenced mainly by clogging in the frontal area because the first layers were fouled significantly more than the deeper layers.

## 1. Introduction

Polymeric hollow fiber heat exchangers (PHFHEs) are an alternative to common metal heat exchangers in low temperature applications. The advantages are the low cost of the material, their low weight and corrosion resistance. Their heat transfer surface consists of hundreds or even thousands of fibers with a small outer diameter, commonly 0.4–1.2 mm, and the wall thickness is about 10% of the diameter. PHFHEs were first presented by Zarkadas in 2004 [1] as an alternative to conventional shell and tube heat exchangers.

The thermal conductivity of polymer materials is much lower than that of metals, which is why the application of polymeric heat exchangers has limited success in comparison to metal ones [2,3]. PHFHEs are very compact heat exchangers that can be used in industry and domestic settings [4]. A general review of polymer heat exchangers is given in [5]. Typically, polymeric heat exchangers are made of tubes with a diameter of 10 mm or more. These tubes have a wall thickness of 1–2 mm and, due to the low thermal conductivity of polymers, the resulting thermal performance of these heat exchangers is poor. Tiny hollow fibers with a wall thickness of about 0.1 mm overcome the disadvantage of polymers’ low thermal conductivity.

The polymeric heat exchangers for liquid-to-gas used in this study were introduced in 2014 [6]. Two types of heat exchangers made of polypropylene hollow fibers (with a wall thickness of 0.05 mm and outside diameters of 0.55 mm and 0.7 mm) with a heat transfer area of 0.2–0.25 m^2^ were studied. The heat transfer performance was studied with hot (40–90 °C) ethyleneglycol-water brine flowing inside the fibers and cooling air flowing across them. The experiments showed that hollow fiber cross-flow heat exchangers can achieve high overall heat transfer coefficients (300–600 W/m^2^·K).

Another study that describes the use of PHFHE in liquid-to-gas application is given in [7,8]. A heat exchanger made of polypropylene fibers was tested in standard conditions for air conditioning, i.e., air temperature of 27 °C and relative humidity of 50%. Dropwise condensation and good condensate removal was observed.

To show the competitiveness of polymeric hollow fiber heat exchangers in relation to metallic finned tube ones, this type of heat exchanger was tested as an automotive radiator [9]. The heat transfer performance and pressure drops were studied with hot (60 °C) ethyleneglycol-water brine flowing inside the fibers and air (20 °C) outside. It was observed that the overall heat transfer coefficients (up to 335 W/m^2^·K), and pressure drops are competitive in relation to conventional aluminum finned tube radiators. All the results given above are for clean heat exchangers and do not provide any details and on the fouling issue.

Air-side fouling of heat exchangers occurs through sedimentation and a deposit of particulate matter from the processed air and as a deposit associated with corrosion. Depending on the nature of the fouling (particle deposit, corrosion and biofouling), the deposit can consist of dust, fibers, products of corrosion or a biological deposit.

Typically, polymeric surfaces are more resistant to biofouling than metal surfaces [10]. The biofilm mass deposited on polymer surfaces is several magnitudes smaller than on stainless steel surfaces. The promising low biofilm formation on the polymers is attributed to the combination of inherent surface properties, roughness, surface energy and hydrophobicity. Polymeric heat transfer surfaces are also preferably used in desalination processes. The main reason is their resistance to corrosion fouling. Due to their chemical stability when exposed to aggressive chemicals, the fouling properties of polymers are considered to be much better than those of steel [11]. The kinetics and quantity of crystallization fouling on different polymeric surfaces were studied with the salts of calcium sulfate and calcium carbonate and compared with those on stainless steel. The promising low scaling affinity of the polymers is attributed to the fact that their surface properties are different to stainless steel.

Particulate fouling mechanisms are described in detail in the monography [12]. Polymeric surfaces are not considered separately, but three principal factors responsible for the adhesion of particles on dry heat transfer surfaces in air are mentioned:

(1) Van der Waals forces of attraction;
(2) Electrostatic forces in systems of oppositely charged surfaces;
(3) The larger the contact area, the greater the total attractive force.

All three of these factors are favorable for polymeric surfaces in comparison to metallic ones. The publication [12] shows that the Lifshitz-van der Waals constant of copper in a vacuum is 8, whilst for magnesium oxide it is 3 and for polystyrene about 2.

Air-side pressure drops and the heat transfer of finned tube heat exchangers with different types of dusts were studied by Bell [13]. The authors found that the deposit has a very significant impact on the pressure drop, increasing the pressure drop of the microchannel heat exchanger by over 200% for a dust injection of 1600 g·m^−2^. They also showed that coils with louvered fins and small fin pitches (less than 2.0 mm) are significantly more sensitive to fouling compared to wavy plate fin heat exchangers with larger fin pitches.

Extensive experimental work on the long-term deterioration in the thermal performance of air conditioner evaporators was performed by Ahn [14]. The authors analyzed 30 heat exchangers, which had been in field operation for up to 7 years and reported a 44% rise in the pressure drop due to a decrease in the amount of hydrophilicity and particle deposits. The associated reduction in the thermal performance was 10–15% for samples used for 7 years.

Despite particulate fouling of membrane hollow fibers used for air filtration being studied in details by Bulejko [15,16], there is no published data describing particulate fouling of polymeric hollow fiber heat exchangers by air dust. Thus, this study was carried out to examine the expectation that there would be high fouling resistance heat exchangers made from polymeric fibers, not tubes and fins med of aluminum.

## 2. Experimental Procedure

### 2.1. Specification of the Tested Polymeric Heat Exchanger

**Figure 1:**
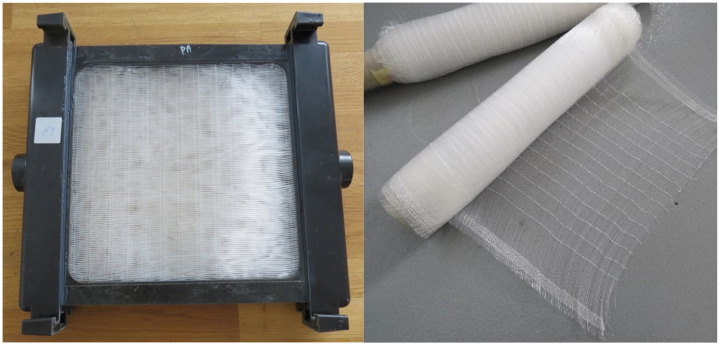
Tested heat exchanger made of polypropylene fiber fabric (**left**) and woven heat transfer surface with separated fibers (**right**).

A heat exchanger made of fiber fabric was tested (see Figure 1). An original approach was utilized to achieve a uniform distribution and separation of fibers—fibers were woven to create a carpet. Fibers with an outer/inner diameter of 0.6/0.48 mm were used, and the fabric density was 55 fibers per 100 mm. The fibers were fixed in frames and formed layers of heat transfer fabric. The heat exchanger air cross-section was 250 mm × 250 mm. There were 140 fibers per layer and in total 14 layers were used. The total fiber number in the heat exchanger was 1960 and the outside surface area was 0.89 m^2^.

**Figure 2:**
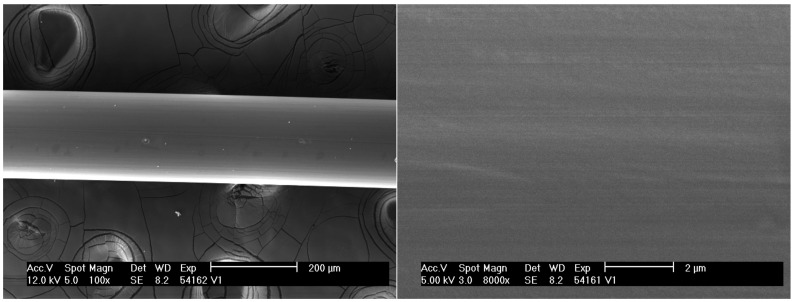
The polypropylene hollow fibers, macroscopic photo with no defects (on the (**left**)) and the surface quality with ×8000 magnification (**right**).

The superior particulate fouling resistance of the hollow fibers was predominantly linked to their surface quality. The hollow fibers were made by extrusion and their final shape was obtained by elongation of the polymeric melt behind the extrusion head. The elongation and solidification of the melt took place concurrently, and the resulting surface of the fiber was very smooth due to the action of the surface tension forces. Figure 2 shows the typical surface quality of an extruded hollow fiber made of polypropylene. The left photo in Figure 1 shows a single fiber with a diameter of 0.6 mm and no macroscopic defects. The right photo shows the details of the surface. It was very smooth and, in the large zoom, there was visible roughness parallel to the fiber axis, which is typical for extruded surfaces.

### 2.2. Specification of Test Dust

Test dust ASHRAE Standard 52.1 (from Powder Technology Inc., Arden Hills, MN 55112, USA [17]) was used for the tests. It is designed for testing filters and heating, refrigeration and air conditioning system components. It has also been used in testing electronic equipment and other industrial and household components. This dust is a good equivalent for residential indoor dust, and it can also approximate exterior dust for condensers installed near fouling sources.

ASHRAE Test Dust 52.1 (see references for the datasheet [18]) is a custom blend of 72% ISO 12103-1. A2 Fine Test Dust, 23% carbon black powder and 5% cotton linters milled in a Wiley Mill fitted with a 4 mm screen. This blend was formulated to specifications given in ANSI/ASHRAE Standard 52.2–2012 and met also the specifications for BS EN 779:2002.

### 2.3. Specification of Testing Equipment and Testing Conditions

**Figure 3:**
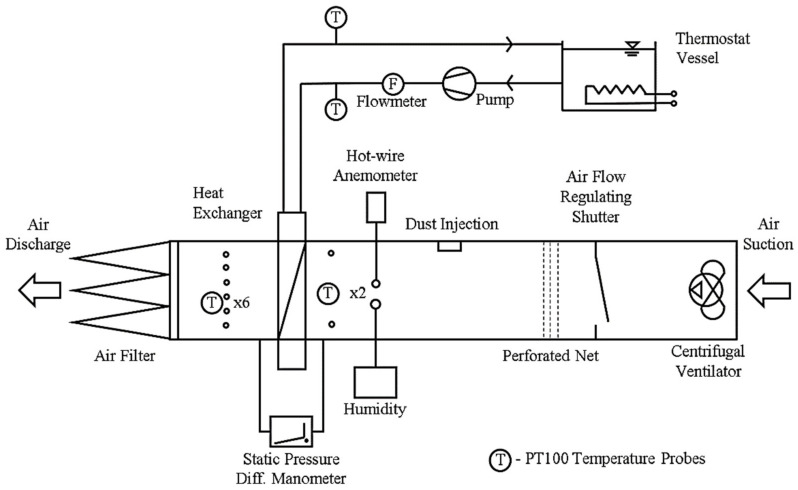
Diagram of the experimental setup.

The diagram of the experiment (Figure 3) shows the water and air circuits. The equipment allows long-term heat transfer tests to be performed with continuous dust injection into the air circuit. The thermal performance data were computed based on the data acquired in the water circuit. The tests were done in conditions typical for the operation regime of indoor HVAC equipment, fan coils and air handling units. The following constant parameters were maintained: air inlet temperature 20 °C, air velocity 1.5 m·s^−1^ (air velocity dropped during tests as fouling increased and was adjusted using a shutter to regulate air flow), relative humidity 20–30%, water inlet temperature 50 °C and water flow rate 430 L/h (this relatively high water flow rate was chosen in order to achieve uniform temperature distribution on the fiber surface).

Test dust was injected into the system twice a day for 10 min periods. Throughout the fouling evolution the air-side pressure drop rose due to the deposits in the air filter and on the heat exchanger, and thus the regulating shutter should be used to maintain constant air velocity of 1.5 m/s.

**Figure 4:**
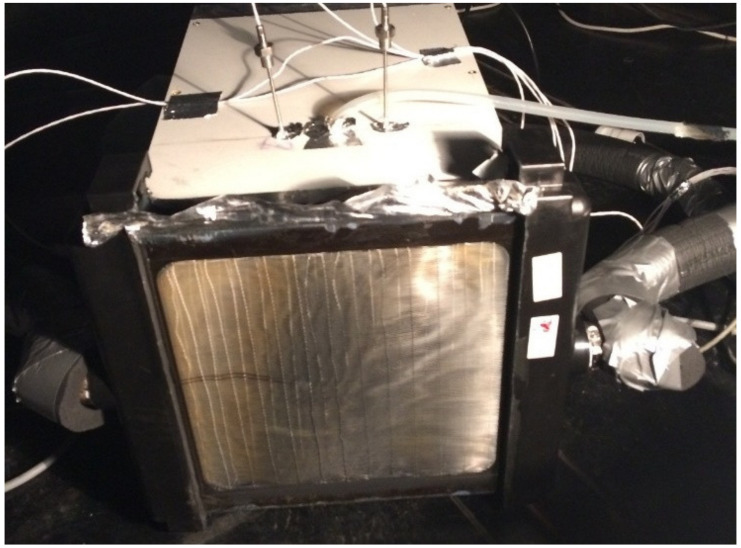
Front side of the heat exchanger connected to the entrance of the wind tunnel after injection of 200 g of the test dust.

A picture showing the heat exchanger connected to the entrance of the wind tunnel can be seen in Figure 4. Connection of air temperature sensors and the pressure connection to the differential manometer can also be seen.

The tested heat exchanger was placed in the wind tunnel and tested for 18 days. For the first 6 days 12.5 g of dust was introduced each day (200 g per m^2^ of frontal area). For days 7–18, the amount of dust was doubled to 25 g a day (400 g per m^2^ of frontal area).

## 3. Results and Discussion

Table 1 includes selected data collected during the experiment. There was air inlet temperature (based on the mean value taken from two sensors); outlet air temperature (based on the mean value taken from six PT100 sensors, Norwalk, CT, USA); air velocity (measured by an anemometer); water flow rate, which was a constant 0.43 m^3^/h, and inlet water temperature, which was a constant 50 °C, and the measured heat transfer rate and pressure drops. All temperatures were measured using PT100 (1/3 DIN Class A precision, Norwalk, CT, USA) sensors with an accuracy of ±0.1 °C. Water flow rate was measured with Krohne Waterflux 3300 flow meter with accuracy ±0.2%. Pressure drop was measured with column manometer MM 200600 (HK Instruments, Keihästie, Finland) with accuracy ±5 Pa. Air velocity was measured with OMEGA HHF-SD1 (Norwalk, CT, USA) hot wire anemometer with accuracy ±10%. Standard deviation of values of heat transfer rate was 5% and pressure drops 10%.

**Figure 5:**
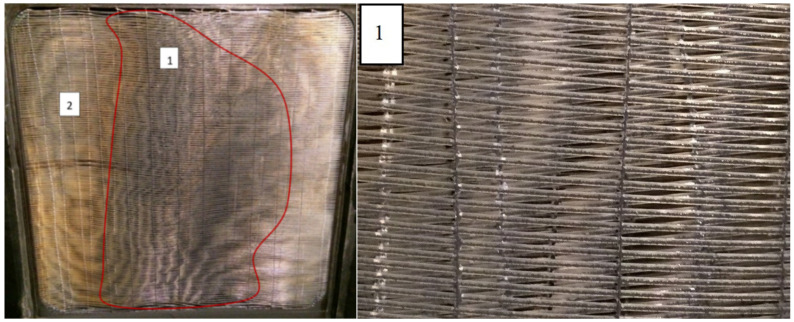
Heat exchanger surface after injection of 400 g m^−2^ of test dust (**left**). There is seen that the fouling is not uniform across the heat exchanger cross-section. Fibers of zone 1 (see magnification on the (**right**)) are more fouled with lot of deposit on the frontal part of the fibers, while the surface of the fibers in zone 2 has only separated particles.

In the following section, some important points of the experiment are described and photographically documented. After 4 days of an experiment and the injection of 400 g m^−2^ of test dust, there are only a small number of separated particles on the fibers (see Figure 5). There was a small reduction in the heat transfer rate (4%) and the pressure drop was not affected. It can be seen that the deposit was not completely uniformly distributed across the heat exchanger frontal area, with the middle (zone 1) more fouled and the rest (zone 2) less. This can be explained by the position of the dust injection window, which is in the middle of the tunnel. Even the 1 m long gap between the injection window and the heat exchanger was not enough to uniformly mix the dust into the airflow.

**Figure 6:**
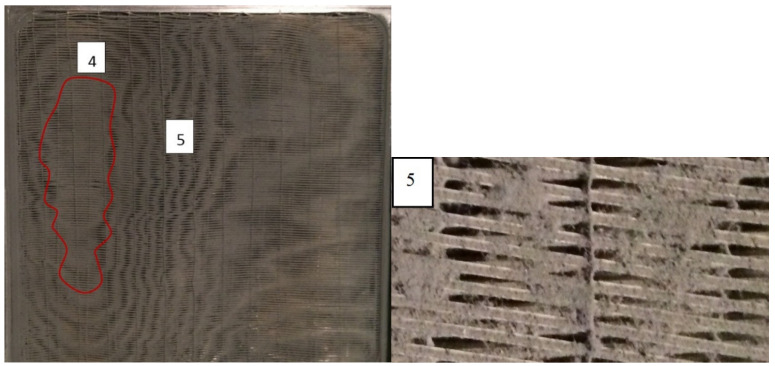
Heat exchanger surface after injection of 1200 g m^−2^ of test dust (**left**). The majority of the front surface of fibers is covered with the deposit layer. There are zones where the deposit blocks the gap between the fibers (as in zone 4) or creates the lines of the blocked zones (as in zone 5, see details (**right**)).

After the injection of 1200 g m^−2^ of test dust, the fibers were covered not by individual particles but by a layer of dust deposit (Figure 6). Fouling was not homogeneous and the deposit evolution was distributed according to some kind of pattern. This pattern may be related to air velocity distribution across the frontal area. There was no additional reduction in the heat transfer or increase in pressure drops comparing to lower loads of the dust. 

**Figure 7:**
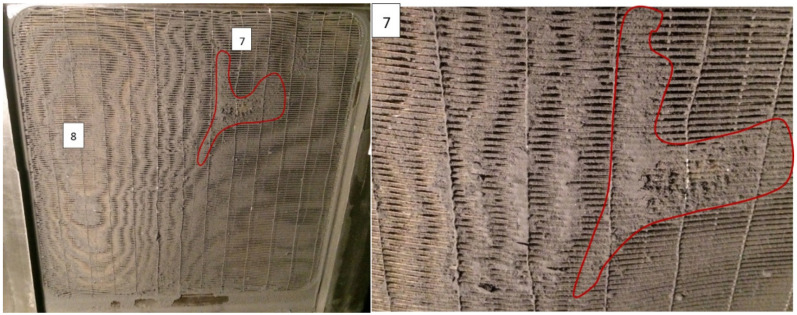
Heat exchanger surface after injection of 2800 g m^−2^ of test dust (**left**); and magnification of the highly fouled zone 7 with complete blockage of the surface (**right**)

After the injection of 2800 g m^−2^ of the test dust, all the fibers were covered not only by separated particles, but by a layer of dust deposit and the fouling pattern can be seen very clearly (see Figure 7). It can be seen that the fouling pattern (see zone 8) corresponded to one found before (zone 4 on Figure 6). There was also a massive dust deposit clogging the frontal area (zone 7). The heat transfer rate was 15% lower and pressure drops increased by 32%.

**Figure 8:**
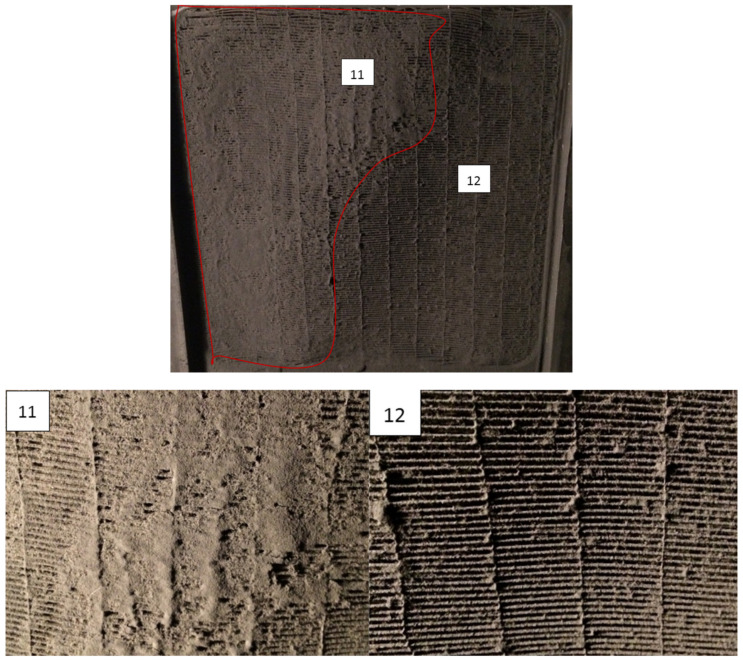
Heat exchanger surface after injection of 4000 g m^−2^ of test dust at the end of measurement. Zone 11 consists of the fibers clogged by the dust, so air cannot flow through. In zone 12, there is a large deposit of the dust on the fibers, but there is no complete clogging of the heat transfer area.

After 18 days of testing, the total dust injection reached 4000 g m^−2^ of test dust and the heat exchanger was massively fouled (38% decrease in heat transfer rate and 91% increase in pressure drops). The experiment was then stopped (see Figure 8). Half of the frontal area (zone 11) was clogged by the dust so air could not pass through. The hollow fibers in zone 12 were covered in dust but air could still flow through. The injection of additional dust would have caused zone 11 to grow, with a very fast rise in pressure drops. In real-life operation of air conditioning there was no regulation of the fan capacity, so the air flow rate would be significantly reduced, causing a deterioration in thermal performance.

**Figure 9:**
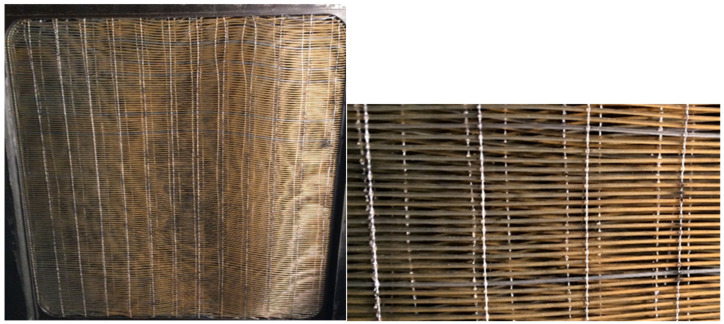
The back of the heat exchanger at the end of measurement. The fiber surface attaches only separated particles with no massive deposit layers.

At the end of the experiment, the frontal area of the heat exchanger was partially blocked by the foulant deposits, but the thermal performance was not so bad. It should be noted that the heat exchanger was formed by 14 layers of fibers. The figure of the back of the heat exchanger after the tests shows that the fiber surface was clean and mostly free of massive deposits of dust particles (see Figure 9). The majority of fouling appeared on the first fiber layers and did not penetrate deeper. It confirms the conclusion that the significant reduction in transferred heat was mainly caused by the clogging of the heat exchanger frontal area. This conclusion was in line with the results found for field operated air conditioner evaporators [14] and for a laboratory tested dry cooler [19]. The fouled samples tested in the wind tunnel [14] show no decrease (in some cases even a small increase due to the more turbulent air flow) in heat output when the air flow was kept at the same level as for clean samples. The results obtained for 15 heat exchangers show that long-term dust particulate fouling influences the thermal performance mainly through the rise of the pressure drop [14]. In the case of the tested dry cooler [15], a 50% increase in air pressure drops was found, and fouling had a slight impact on the thermal performance.

**Figure 10:**
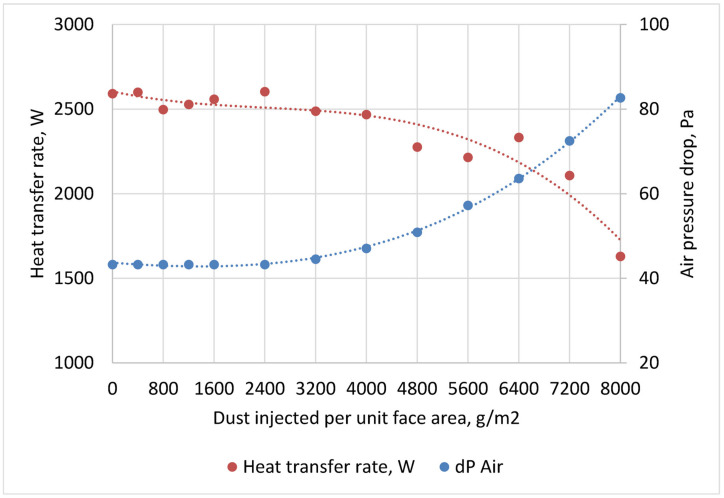
Influence of fouling evolution on heat transfer and pressure drops.

There is pronounced initiation period on the record of the time evolution of pressure drop and heat transfer rate (see Figure 10). The first six days of the test (injection of 800 g/m^2^) provided data in which no fouling effect can be observed. After a certain critical point (approximately 2400 g/m^2^ of test dust) the influence of fouling developed significantly faster.

**Figure 11:**
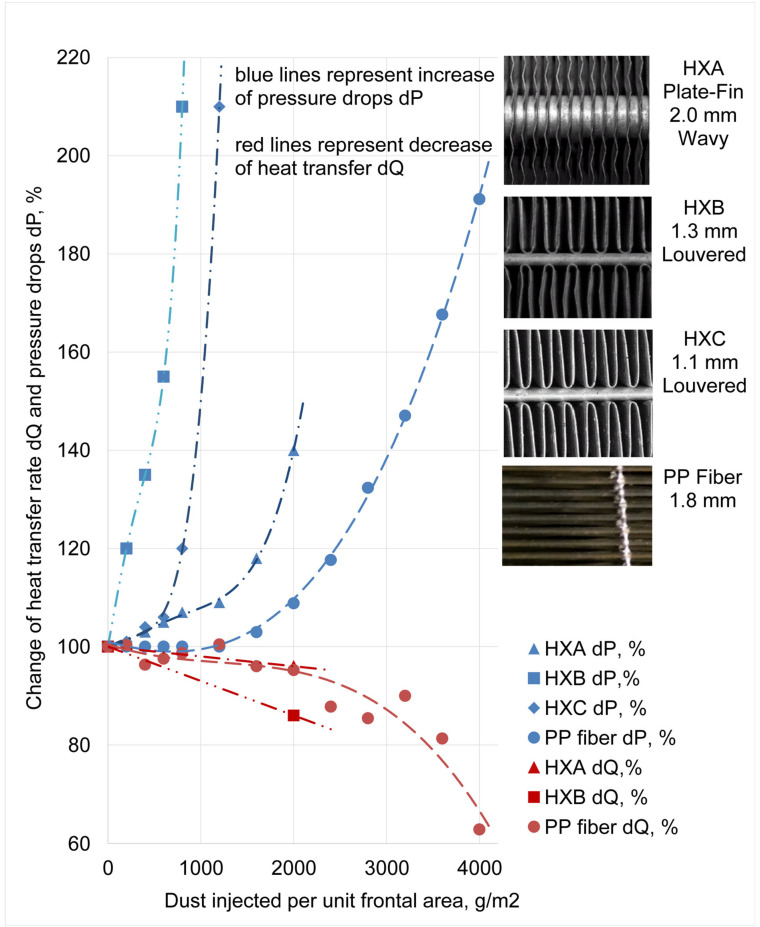
Comparison of fouling characteristics of finned tube heat exchangers HXA. HXB and HXC and polymeric hollow fiber heat exchanger. Data for HXA. HXB and HXC are adopted from Bell and Groll [13].

The fouling evolution results evaluated for the smooth polymeric hollow fibers in this study were compared with the data published for classical metal heat exchangers published in [15]. The first metal heat exchanger was made of copper circular tubes with thin aluminum wavy fins. These heat exchangers were extensively used in HVAC equipment as air heaters/coolers, evaporators and condensers. The second metal type was the heat exchanger with louvered fins. This type of fins was connected by brazing them to the flat aluminum tubes. This type is widely used as automotive radiators. Figure 11 shows a comparison of pressure drops and the performance for metal and polymeric heat exchangers. The significantly better antifouling characteristics of the polymeric heat transfer surface can be observed. The amounts of dust that cause an increase of 50% in the pressure drop was 550 g/m^2^ and 1000 g/m^2^ respectively for the two sizes of louvered heat exchanger. For the plate-fin heat exchanger it was 2100 g/m^2^ and for the polypropylene one it was 3200 g/m^2^. 

The tested heat exchanger was cleaned with a vacuum cleaner after the experiment. Cleaning procedure was performed to check if the deposit is attached to the surface strongly or can be removed with industrial vacuum cleaner—as it is practiced with field operated equipment. As it was expected, the deposit was completely removed and practically no particles remained on the surface. No evidence of any damage or defects was found on the surface too. Thermal performance was not retested after the cleaning, because visual inspection was considered sufficient to confirm that the cleaning recovers the thermal performance to its initial state.

## 4. Conclusions

Particulate fouling evolution on plastic heat exchangers was experimentally studied and evaluated in the presented study. ASHRAE test dust 52.1 was used as the fouling agent in the test because this type of dust best represents the typical fouling conditions found on HVAC and automobile radiators operating in cities. The presence of cotton linters in the dust (5%) was found to be crucial for fouling evolution. The cotton linters initiate the fouling and have the highest impact on rise of pressure drops. Fouling evolution is not uniform with time and the amount of dust injected. It progresses very slowly at the beginning (showing no effect on heat transfer and pressure drops) and the initiation period lasts approximately until the injection of 800 g/m^2^ of dust.

The tested heat exchanger was formed by 14 layers of hollow fibers with an outer diameter of 0.6 mm and fiber spacing of 1.8 mm. The decrease in the heat transfer rate of the heat exchanger was mainly caused by the clogging of the frontal area. It excluded a significant part of the heat exchanger from the heat transfer process because air could not flow through the clogged part. It caused a significant increase in pressure drops as well. The backside layers of the heat exchanger did not show large deposits of dust after the test was completed.

The smooth polymeric surface shows significantly better antifouling characteristics than the classical metal heat transfer surfaces with a similar density. In comparison to the metal louvered heat exchangers, they experienced a 50% increase in pressure loss after three times more test dust was injected. Comparison with the plate-fin metal heat exchanger (fin spacing 2 mm) showed a 50% longer service time of the polymer heat exchanger for an identical rise in pressure drops. 

While single fibers are highly protected from fouling due to the superior surface properties of the polymeric material, the fouling of dense arrangements of fibers can be more pronounced due to clogging in the gaps between them. Larger pitches (2 mm or more) between the fibers are recommended to prevent particulate fouling evolution in very dusty conditions.

## Figures and Tables

**Table 1:** Review of experimental data.

Day	Injected Dust Mass, g	Relative Dust Mass, g m^−2^	Air Inlet T, °C	Air Out T, °C	Air Speed, m/s	Water Outlet T, °C	Heat Transfer Rate Q, W	dp, Pa
1	0	0	19.3	42.9	1.50	44.9	2592	43
2	13	200	19.2	42.9	1.50	44.8	2600	43
4	25	400	19.3	42.9	1.45	44.9	2497	43
5	38	600	19.2	43.1	1.45	45.0	2528	43
6	50	800	19.2	43.0	1.49	45.0	2558	43
7	75	1200	19.5	42.4	1.57	44.8	2604	43
8	100	1600	19.4	42.5	1.49	45.0	2488	45
9	125	2000	19.3	42.7	1.46	45.1	2468	47
10	150	2400	19.3	43.1	1.30	45.3	2276	51
12	175	2800	18.7	43.4	1.31	45.5	2214	57
13	200	3200	21.5	43.5	1.35	45.6	2332	64
16	225	3600	20.5	44.1	1.27	46.0	2108	73
18	250	4000	19.5	45.4	1.07	46.7	1629	83
